# Antisense Transcription in Plants: A Systematic Review and an Update on cis-NATs of Sugarcane

**DOI:** 10.3390/ijms231911603

**Published:** 2022-10-01

**Authors:** Luciane Santini, Leonardo Yoshida, Kaique Dias de Oliveira, Carolina Gimiliani Lembke, Augusto Lima Diniz, Geraldo Cesar Cantelli, Milton Yutaka Nishiyama-Junior, Glaucia Mendes Souza

**Affiliations:** 1 Departamento de Bioquímica, Instituto de Química, Universidade de São Paulo, São Paulo 05508-900, Brazil; 2 Laboratório de Toxinologia Aplicada, Instituto Butantan, São Paulo 05503-900, Brazil

**Keywords:** plants, asRNA, cis-NAT, systematic review, artificial antisense, transgenic plants, sugarcane

## Abstract

Initially, natural antisense transcripts (NATs, natRNAs, or asRNAs) were considered repressors; however, their functions in gene regulation are diverse. Positive, negative, or neutral correlations to the cognate gene expression have been noted. Although the first studies were published about 50 years ago, there is still much to be investigated regarding antisense transcripts in plants. A systematic review of scientific publications available in the Web of Science databases was conducted to contextualize how the studying of antisense transcripts has been addressed. Studies were classified considering three categories: “Natural antisense” (208), artificial antisense used in “Genetic Engineering” (797), or “Natural antisense and Genetic Engineering”-related publications (96). A similar string was used for a systematic search in the NCBI Gene database. Of the 1132 antisense sequences found for plants, only 0.8% were cited in PubMed and had antisense information confirmed. This value was the lowest when compared to fungi (2.9%), bacteria (2.3%), and mice (54.1%). Finally, we present an update for the cis-NATs identified in *Saccharum* spp. Of the 1413 antisense transcripts found in different experiments, 25 showed concordant expressions, 22 were discordant, 1264 did not correlate with the cognate genes, and 102 presented variable results depending on the experiment.

## 1. Introduction

Although most of the eukaryotic genome has been transcribed, a tiny part encodes proteins [1,2]. Besides the protein-coding genes and the well-studied regulatory RNA-coding genes, almost the entire eukaryotic genome has occasionally been transcribed, albeit in low amounts [3]. Most of these non-canonical transcripts are yet to be deeply investigated, especially in plants.

**Figure 1:**
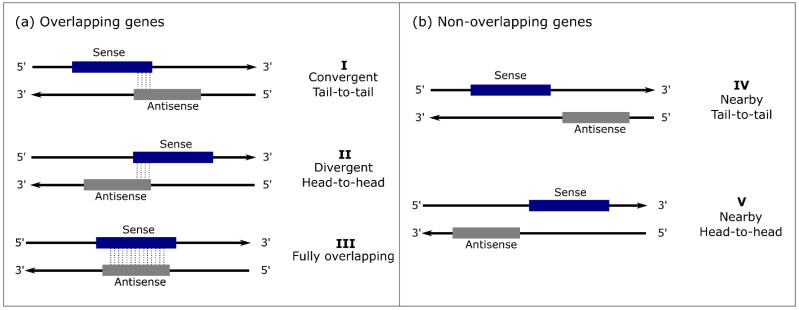
Schematic representation of the five main classes of cis-NATs adapted from Osato et al. [17]. Sense genes are presented in blue and antisense in gray. (**a**) Classes of the cis-NAT with overlapping genes: (**I**) “Convergent or tail-to-tail” represents overlapping genes connected via their 3′ UTRs; (**II**) “divergent or head-to-head” consists of overlapping genes connected by their 5′ UTRs; (**III**) “fully overlapping” represents cis-NAT completely overlapping the sense gene in the opposite strand. (**b**) Classes of the non-overlapping cis-NATs: (**IV**) “nearby tail-to-tail” when the 3′ UTR of one gene is close to the 3′ UTR of the other gene; and (**V**) “nearby head-to-head” when the 5′ UTR of one gene is close to the 5′ UTR of the other gene.

With the advancement of genomics and transcriptomics in the last few decades, unprecedented data have been obtained, such as the 1000 Plant Genomics and Transcriptomics Initiatives [4,5,6]. The revealed genes and transcripts are usually included in the NCBI [7] or plant-specific databases such as Phytozome [8], TAIR [9], and SUCEST-FUN (https://sucest-fun.org), providing information for gene expression studies. New transcripts are continually being documented and characterized, fast expanding the list of known RNAs. Several of these RNAs seem to have regulatory roles, positively [10,11] or negatively [12] regulating the expression of other genes, such as the natural antisense transcripts (NATs). NATs are transcribed in the opposite orientation and are complementary to the sense transcripts (with mismatches or perfect matching) in the same (cis-NAT) or another locus (trans-NAT) [13]. In general, cis-NATs are easily identified in transcriptome studies, and most of the well-known NATs belong to this class. Cis-NATs are widespread in eukaryotes, and their observed frequency in human, *Drosophila melanogaster,* and *Arabidopsis thaliana* genomes was found to be 4–9%, 22%, and 10–20%, respectively [13,14,15,16]. The cis-NAT classification is based on their relative orientation and overlap with the sense gene and may vary according to different authors. The five main classes defined by Osato [17] are considered here, namely (I) “convergent or tail-to-tail”; (II) “divergent or head-to-head”; (III) “fully overlapping”; (IV) “nearby tail-to-tail”; and (IV) “nearby head-to-head” (Figure 1). The occurrence and the overlapping type might be considered to define the assertive approach for cis-NAT identification. Reis and Poirier [13] recently proposed a framework for identifying cis-NATs from classes I to III, considering the correlation between the antisense and its cognate gene expression and its subcellular location.

Other aspects should be considered in cis-NATs studies such as the coding capacity of the cognate loci and its length. NATs may be transcribed either from protein-coding or non-protein-coding regions [18,19] and are usually included in more general classes of transcripts. NATs up to 200 nucleotides are classified as short non-coding RNA (sncRNA), whereas the longer ones are long non-coding RNA (lncRNA) [18]. It is currently estimated that over 90% of cis-NATs detected in plants are lncRNA [13]. Several approaches have been used to investigate the different classes of NATs and are briefly presented here (Box 1).

NATs can be involved in either transcriptional or post-transcriptional regulation. They were initially considered negative regulators of cognate genes [20]. However, the antisense regulatory function seems to be more complex, acting in some cases as a positive regulator of gene expression by enhancing translation [10]. According to their expression levels, sense/antisense (SS/AS) pairs are classified into concordant or discordant expressions, respectively, whenever they exhibit positive or negative correlations. For instance, in *Arabidopsis*, it was reported that most NATs exhibit concordant expressions [21]. A total of 21 NATs were selected for gene silencing experiments and, in line with the findings mentioned above, most sense counterparts (15) were also found to be downregulated upon NAT knockdown. In contrast, only a few of them were found to be upregulated (3) or with no significant changes in their expression level (3). Paradoxically, collision from RNA Pol II from SS/AS pairs at the same locus is thought to result in the abortion of transcription [22], contradicting the observation that most NATs are positively correlated with the corresponding sense transcript. This apparent contradiction seems to be resolved by differential expression from different alleles and/or differential expression at the individual cell level, with trans factors playing additional roles that culminate in the concordant expression [23,24]. NATs can alter chromatin structure and accessibility as they recruit protein complexes involved in histone modification [21,23,25]. In post-transcriptional regulation, one instance of siRNA formation from NAT induced by salt stress was described in *Arabidopsis* [26].

Despite antisense transcription being first reported in a virus more than 50 years ago [27] and in plants more than thirty years ago [28], the knowledge of these transcripts and their functions is mainly related to cis-NAT and remains incipient [13,29].

These transcripts have been identified in several plant species playing different biological roles, for instance, in plant development or response to biotic or abiotic stresses [21,23,30,31,32,33]. However, most of the cis-NAT identified in plants must be characterized. In this context, our objectives are to present a systematic review of asRNA in plants, quantify the articles published up to 2021 and the antisense genes available in the NCBI database, and update this scenario for the published data on sugarcane.

Box 1 **Techniques to study antisense transcripts.**

Like other transcripts, NATs can be identified at the individual gene level or the genome-scale level. For the screening of target genes, RT-PCR, RT-qPCR [34,35], dot-blot hybridization [36], and Northern blot [37] techniques can be used. The strand specificity of the cDNA in RT-PCR is essential for sense and antisense transcript detection. The use of different RT primers, i.e., reverse PCR primer for the sense strands and forward PCR primer for the antisense strand amplification. The main high-throughput approaches that have been used are SAGE (Serial Analysis of Gene Expression)-derived techniques [38,39,40,41,42,43], microarrays [44,45], and tiling arrays [46,47,48,49] or strand-specific RNA sequencing [50,51,52,53,54,55]. The details and usual procedures of those techniques have been reviewed [56,57,58]. Until recently, strand-specific arrays were the standard techniques for detecting and studying antisense transcripts in plants. Lately, RNA-Seq has become a widely adopted technique to study transcripts at the genome-scale level. Strand-specific protocols that keep track of strand orientation are necessary for detecting NATs. After reads have been mapped to a reference genome, transcripts can be classified as antisense by a program (e.g., gffcompare, cuffcompare [59], toRNAdo [60]) that systematically verifies if detected read overlaps with annotated genes that are transcribed from the opposite strand. Finally, the protein-coding potential must be assessed by an appropriate program (e.g., CPAT [61]) to distinguish between coding and non-coding RNAs.

Other sequencing-derived techniques targeting nascent RNAs have been used to successfully detect NATs [62,63,64,65,66], such as the global run-on sequencing (GRO-seq) [63,67], native elongating transcript sequencing (NET-seq) [64,67], and sequencing of short metabolically labeled RNA [65] that was used to investigate unstable RNAs in *Arabidopsis*. With these methodologies, it is possible to select the nascent RNA, which is purified, sequenced, and mapped to the genome. A high-resolution map of *Arabidopsis thaliana* nascent transcripts showed that RNA Polymerase occupied ~4% of the nuclear genome in the antisense strand of the gene [63]. Szabo et al. [65] compared steady-state and nascent 5-EU-labeled RNA (Neu-seq) libraries. They found a significantly higher antisense detection by Neu-seq, indicating that sequencing approaches targeting unstable RNAs are preferable in this type of study. NAT can also be investigated at the translational level using polysome- or ribosome-profiling approaches [19,68].

The unprecedented amount of sequencing data has allowed some groups to use datasets already available to identify antisense transcripts [31,59,69]. The functional role of antisense lncRNA can be studied using loss-of-function tools and/or combining multiple molecular and cellular techniques. Transcript silencing using siRNA has been used but with some unsuccessful results mainly due to the low expression of lncRNA and its main localization [70]. A database of validated “Clustered regularly interspaced short palindromic repeats-associated protein 9” (CRISPR/Cas9) single guide RNAs for lncRNAs was created based on the curation of more than 200 published articles. However, it was possible to include only one plant lncRNA, lncRNA1459 from *Solanum lycopersicum* [71], which regulates tomato ripening [72]. Esposito and colleagues reviewed studies using the CRISPR-CAS9 genome editing tool as a screening method to identify functional lncRNA in cancer proliferation and drug resistance [73]. The continuous improvement of this technique and the expansion of its use in plants may help its application for screening lncRNA in plants. The combination of RNA fluorescence, in situ hybridization, and single-cell transcription kinetics quantification in *Arabidopsis* cells with or without the COOLAIR, a cold-induced long antisense intragenic RNA, showed that the antisense expression modulates the cell size dependency of the sense transcription [74]. A study of DNA (de)methylation in response to hyperosmotic stress in *Arabidopsis* showed that antisense lncRNAs are regulated by this stress and mediate the effects of stress-inducing differentially methylated regions (DMR) [75].

## 2. Methods

### 2.1. Plants asRNA Systematic Review

**Figure 2:**
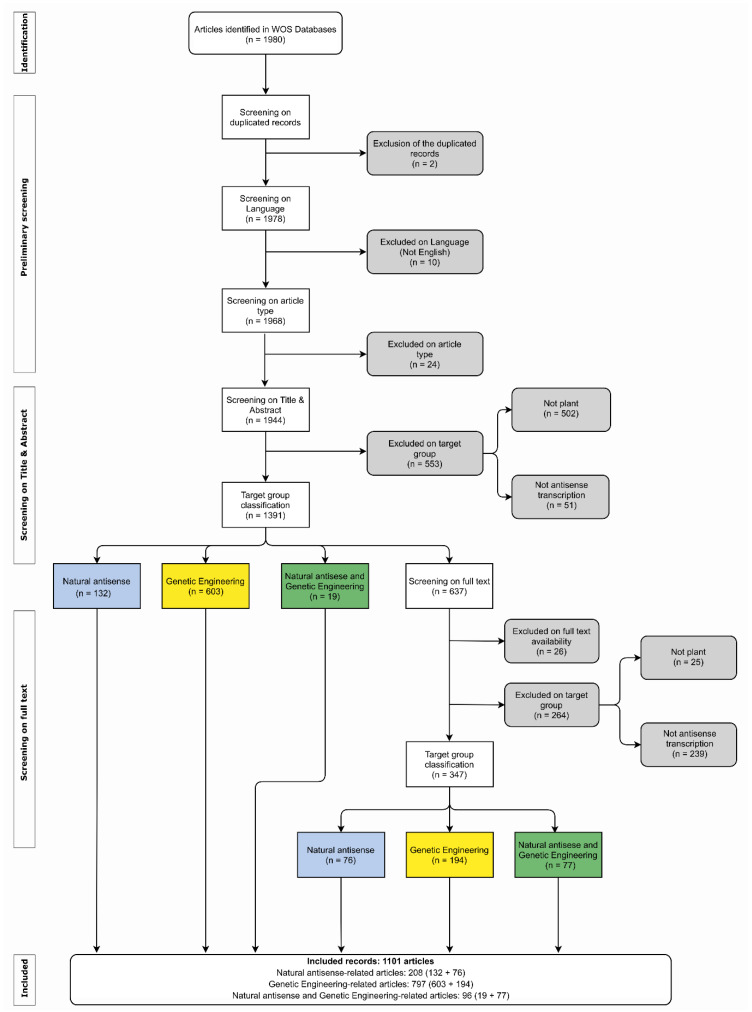
Systematic review workflow and results of article categorization. White: screening process; gray: excluded records; blue: articles categorized as “Natural antisense”; yellow: articles categorized as antisense expression used in “Genetic Engineering”; green: articles classified as “Natural antisense and Genetic Engineering”.

To further our knowledge of asRNA, we conducted systematic searches in the Web of Science (WOS) and NCBI databases for papers and sequences investigating the scientific publications associated with genomic sequences related to asRNA currently available for land plants. The systematic review of publications was performed in all databases of the Web of Science using the Advanced Search tool with the following string (TS = ((“antisense RNA” OR “antisense transcript” OR “natural antisense” OR “asRNA” OR “antisense expression”) AND plant*)). All years were selected for the timespan and English was defined as the search language. The first search was conducted on 5 April 2020 and was updated monthly. The last update was performed on 18 February 2022, considering only the articles published up to 2021. Obtained records were filtered by document type and the documents classified as patent, meeting, abstract, biography, and retracted publication were excluded. The references were exported in “ris” format to the EPPI-reviewer software [76,77] and were manually categorized. The strategy based on the PRISMA (Preferred Reporting Items for Systematic reviews and Meta-Analyses) guidelines [78] is summarized in Figure 2. The included records were classified as “Natural antisense”, “Genetic Engineering”, or “Natural antisense and Genetic Engineering”-related publications.

The systematic search for gene sequences related to antisense expression started with an advanced search in the Gene database from NCBI (https://www.ncbi.nlm.nih.gov/), using the following string: plants [organism] AND (“antisense RNA” OR “antisense transcript” OR “natural antisense” OR “asRNA” OR “antisense expression”). This search was conducted on 18 February 2022. All genes were submitted to the classification workflow (Figure S1). First, gene records associated with citations were obtained by the “PubMed” standard filter. All the genes cited in PubMed were manually checked for antisense transcription evidence based on (I) gene description; (II) graphical representation of the antisense alignment; and (III) information available in the related articles. We performed similar searches for fungi, bacteria, and mice to compare the availability of the antisense sequence information in the “Gene” database for each organism.

### 2.2. Sugarcane asRNA

In order to update the antisense expression scenario in sugarcane, we explored transcriptomic data from multiple published experiments. Eight published experiments (Table 1) used the same oligo array [44] to study the gene expression of different sugarcane genotypes under greenhouse [44,79,80,81] or field conditions [33,82,83,84] and treatments such as drought and ethylene pulverization. This customized oligo array (CaneRegNet—Agilent Technologies) includes 21,901 probes in duplicate, which represent 14,522 different sugarcane-assembled sequences (SAS) from the SUCEST database (Sugarcane Expressed Sequence Tag project) [85]. Among the probes, 7380 were designed to hybridize into antisense transcripts.

We used the expression values from each mentioned experiment (Table 1) available at the SUCEST-FUN database (https://sucest-fun.org/wsapp/, accessed on 6 March 2021) to investigate the expression levels of antisense transcripts and their cognate SAS identified in sugarcane leaves. The list of significantly expressed genes and their respective expression value were downloaded using the “Search Significant Expressed Genes” tool (https://sucest-fun.org/wsapp/searchSignificantExpressedGenes.do, accessed on 6 March 2021) in the “Cane Gene Expression” module. The following projects were selected: CaneRegNet Ethylene, CaneRegNet growth and maturation, CaneRegNet1 Ancestral, CaneRegNet1 Circadian, CaneRegNet1 Drought, and FCaneRegNet1 Drought. The expression value was obtained for the biological replicates by selecting the option “Expressed Genes by Crossing”.

All data were filtered to keep genes with probes designed to capture the expression on both sense and antisense orientations and with expression evidence on leaf samples in all biological replicates evaluated in each experiment. SAS without antisense expression or with evidence detected only in one biological replicate were not considered.

Filtered data were used to investigate global and gene-specific expression patterns in two scenarios: when only sense transcripts were expressed and when both sense and antisense transcripts were expressed in the same experiment. Finally, the global expression analyses of the SS/AS pairs were performed for each experiment, aiming to identify the expression profile of the sense transcripts in the presence and absence of the cognate antisense expression. The differences in expression values were verified by a *t*-test (0.05) corrected by FDR (false discovery rate).

All filtering, data plotting, and analyses were performed using packages contained in the R version 4.0.3 software [86] according to the scripts available in the GitHub repository (https://github.com/sucestfun/Sugarcane_Antisense_Expression, created on 15 August 2022).

## 3. Results and Discussion

### 3.1. Plants as RNA Systematic Review

**Figure 3:**
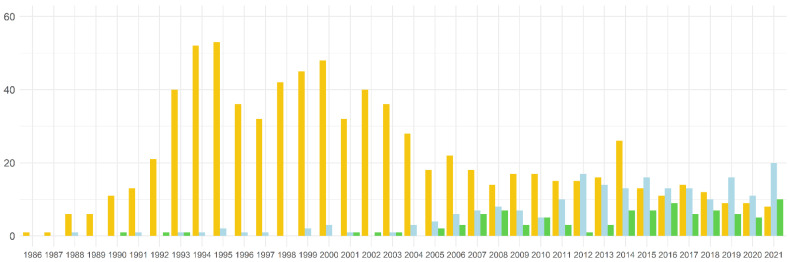
Numbers of antisense transcription-related articles included in Web of Science databases. Barplot distribution of records according to year of publication and the target group categorization implemented in the systematic review workflow. Blue: “Natural antisense”-related articles; yellow: articles categorized as antisense expression used in “Genetic Engineering”; green: articles included in both categories “Natural antisense” and “Genetic Engineering”.

A total of 2371 records were obtained from the Web of Science databases and submitted to filtering by document type. This first filtering step classified 391 records as patent, meeting, abstract, biography, or retracted publications. The exclusion of these records resulted in 1980 articles being manually screened according to the systematic workflow (Figure 2). The classification procedure resulted in 1,101 articles being classified as “Natural antisense”-related (208), artificial antisense used in “Genetic Engineering” (797), or “Natural antisense and Genetic Engineering”-related publications (96). Our analysis confirmed that 56.6% of the articles (1101/1944) were related to antisense transcription in plants. The oldest publications related to antisense transcription in plants and available in the WOS databases were from the 1980s (Figure 3; Tables S1–S3).

Initially, most of the articles on antisense in plants were dedicated to exploring the complementarity of the sequences in Genetic Engineering as a tool for gene silencing in several species [86,87,88,89,90,91,92,93]. A significant increase in this type of publication was observed in the 1990s, with more than 50 published articles in 1994 and 1995. The first paper on NAT in plants was published in 1988 [28]. However, a sharp increase occurred after 2005 due to the greater availability of omics data in the 2000s [94]. An upward trend in the publication of NAT-related articles has been identified in the last decade, surpassing 20 publications in 2021. Articles classified in both categories that have been published since 1990 include reviews and articles using the NAT sequences in Genetic Engineering (Figure 3). All NAT and NAT and Genetic Engineering-related articles (304) were checked for the type of publication (Table S4) and plant species they focused on (Tables S1 and S2). Most of these publications were research articles (76%; 231/304), followed by reviews (23%; 70/304) and book chapters (1%; 3/304) (Table S4). Regarding the classification by species, 70 plant species were studied in these articles. However, only three species were represented in 45.5% of publications, *Arabidopsis thaliana* (33%; 111/304), *Oryza sativa* (9.5%; 29/304), and *Zea mays* (3%; 10/304). These data indicate that the knowledge about antisense in non-model plants is still limited.

Although most of the natural antisense detected in plants have not been characterized, we found that antisense transcription in response to myriad stimuli was briefly exemplified. Matsui et al. [47] detected antisense transcription in *A. thaliana* under stress caused by temperature, drought, salinity, and ABA treatment. Heat-responsive antisense transcripts were found in *Brassica rapa* using RNA-seq and small RNA (sRNA) deep sequencing approaches [30]. Otherwise, NATs were detected by deep sequencing in *Manihot esculenta* and *Ricinus communis* under chilling treatment [95]. Cold-responsive antisense transcripts were also identified by sequencing in *Solanum lycopersicum* [96], *B. rapa* [97], and *A. thaliana* [98,99].

Light conditions also affect antisense transcription in plants. *Oryza sativa* subjected to continuous dark treatment showed a decrease in the catalase gene (*CatB*) expression in roots caused by an accumulation of *CatB* sense and antisense unspliced transcripts, detected by RNA dot-plot hybridization [36]. In contrast, Tiwari et al. [100] used strand-specific RNAseq to investigate small RNA induced by high light acclimation in *A. thaliana* and detected NATs and double-strand RNAs derived from NATs.

Drought-responsive NATs were detected via oligo arrays in *A. thaliana* [47] and *Saccharum* spp. [44] and via sequencing approaches in *Musa* spp. [101], *Gossypium hirsutum* [102], *M. esculenta* [103], *Zea mays* [104], *Populus trichocarpa* [105], *Solanum lycopersicum* [106], and *Oryza nivara* [107].

Natural antisense transcription was also associated with the response of plants to chemical stress such as mercury (*Medicago truncatula*) [108], cadmium (*O. sativa*) [109], boron (*Z. mays*) [110], and methyl methane sulfonate (*A. thaliana*) [111]. Additionally, salt-response NATs were observed in *M. truncatula* [112], *Z. mays* [110], and *Glycine max* [112], using RNA sequencing techniques.

Concerning biotic stress, NATs were identified in several plant–pathogen interactions, such as *O. sativa* in response to *Magnaporthe grisea,* whose transcriptional profile was investigated using RL-SAGE (Robust Long SAGE) methodology [113]. Other fungi response NATs were found by RNA sequencing in *Vitis pseudoreticulata* and *V. quinquangularis* [114], *Brassica napus* [115], and *V. vinifera* [116]. Muthusamy et al. [117] sequenced the transcriptome of *Musa* spp. in response to *Mycosphaerella eumusae* and *Pratylenchus coffeae* and found several differentially expressed NATs in resistant and susceptible genotypes.

The *TalncRNA73* detected in a suppression subtractive hybridization (SSH) library in wheat in response to rust is an antisense of a hypothetical protein [118], whose function remains unknown [119]. In *Malus* × *domestica*, NATs were found in the ASGV (apple stem grooving virus) infection transcriptome [120].

Furthermore, Wang et al. [121] used RNA sequencing to investigate herbivore-elicited lncRNA in *O. sativa*. They observed a significant increase in *NATJAZ10* expression in response to herbivory, concomitant with the upregulation of its cognate gene, *JAZ10* (Jasmonate-zim-domain protein 10).

Reproduction events in plants may be affected by antisense transcription. Several differentially expressed NATs were detected during maize flowering [122], as well as in the different development stages of florets of apomictic and sexual *Paspalum notatum* [123]. Moreover, an antisense transcript of the mitochondrial rice *atp*6 gene may be involved in cytoplasmic male sterility (CMS) [124].

Organelle-specific antisense transcription has also been observed in plants. Ruwe et al. [125] identified NATs in the chloroplast and mitochondrial transcriptomes of *A. thaliana*. Similarly, NATs have been found in the chloroplasts of *Salvia miltiorrhiza* [126].

Evidence of antisense regulation in cellular programming and differentiation [52,127], circadian rhythm [49], and growth and maturation [33] reveals that NATs play fundamental roles in all biological processes.

**Figure 4:**
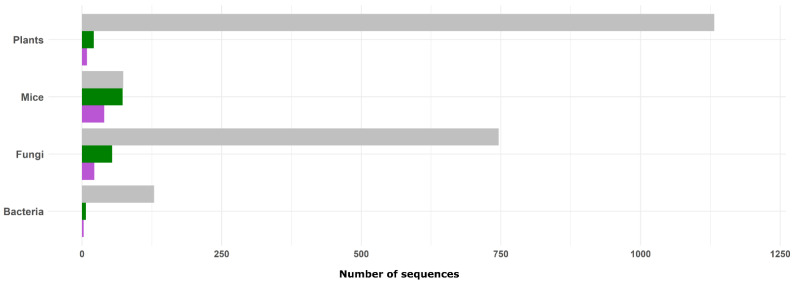
Antisense-related genes available for plants, mice, fungi, and bacteria in the Gene Database from NCBI. Recovered sequences in an advanced search using the string [organism] AND (“antisense RNA” OR “antisense transcript” OR “natural antisense” OR “asRNA” OR “antisense expression”). Available sequences (gray); sequences cited in PubMed articles (green); sequences with verified antisense transcription information (purple). The search was conducted in the Gene database from NCBI (https://www.ncbi.nlm.nih.gov/) on 18 February 2022.

The systematic search for gene sequences related to antisense expression in plants resulted in 1,132 genes (Figure S1a and Figure 4). Only 21 of these genes have been cited in publications and the antisense transcription was confirmed for nine of them (Figure 4; Table 2). The remaining 12 genes cited in PubMed represent spurious results due to the word antisense in the complementary information on the gene description web page, in the title, or in the abstracts of articles citing such genes.

Compared to the other investigated organisms, the highest number of gene sequences was obtained for plants (Figure S1a and Figure 4). However, only 0.8% (9/1,132) of these genes were confirmed as being antisense-related (Table 2). Six genes were identified in *A. thaliana* and the most cited was the *MIR398b* (AT5G14545) with seven citations in PubMed (Table 2). The overexpression of this microRNA inhibits the expression of its cognate gene *AtC2GnT* (AT5G14550) and increases the susceptibility of *A. thaliana* to *Phytophthora parasitica* [128]. Another two *MIR389* genes were identified in this search, the MIR389c from *A. thaliana* and the *MIR389* from *Brassica rapa*, with four and three citations in PubMed, respectively (Table 2).

Another relevant antisense gene detected in this systematic search was the *COOLAIR* (AT5G01675), responsible for repressing the *FLOWERING LOCUS C* (*FLC)* during vernalization in *Arabidopsis* [129,130,131]. The sense *FLC* (AT5G10140) is a well-studied antisense-regulated gene with 142 citations in PubMed (Table 2).

The five remaining antisense genes were detected in *A. thaliana* [132,133], *Solanum lycopersicum* [134], and *Zea mays* [135]. They have been cited once in PubMed and their putative cognate genes are protein-coding (Table 2).

The low availability of well-characterized antisense sequences reinforces the idea that the high-throughput data generated for plants remains understudied. Mice represented the lowest number of available sequences (74) (Figure S1d); however, they were the best characterized (Figure 4; Table S5). As mice are models for human studies, efforts have been devoted to accurately characterizing their genes. Of 74 mice putative antisense-related genes, 98.6% (73/74) were cited in PubMed, and 54.1% (40/74) were confirmed as antisense. Twenty-three mice antisense genes had more than ten PubMed citations, with the most cited being the *Kcnq1ot1* (Table S5). This gene regulates several microRNAs that play essential roles in the cell inflammatory response [136], cerebral ischemia and reperfusion injury [137], diabetic cardiomyopathy, and other diseases [138].

Sequences of fungi and bacteria have also been poorly studied. Of 746 putative antisense genes found for fungi, 7.2% (54/746) were cited in PubMed and 2.9% (22/746) were confirmed as antisense (Figure S1b; Table S6). However, the cognate genes were identified only for eight antisense genes from *Saccharomyces cerevisiae* and one from *Schizosaccharomyces pombe*. The 13 remaining genes were identified in *S. pombe* as predicted antisense non-coding RNAs [139]. These sequences have a provisional status and need to be characterized (Table S6).

Among the four groups of organisms investigated here, bacteria presented the second lowest precision in the antisense description (Figure 4). Only 5.4% (7/129) of the putative antisense genes were cited in PubMed and 2.3% (3/129) were antisense-confirmed (Figure S1c; Table S7).

The *Escherichia coli micA* gene was the most cited bacterial antisense gene with 25 related articles in PubMed (Table S7). This gene stands out for being a post-transcriptional regulator of several genes [140,141,142] and for acting in the mechanisms of virulence [143]. A vaccine produced with *micA*-derived OMVs (outer membrane vesicles) protected mice against *Salmonella typhimurium* [143].

Our results show that information about antisense genes deposited in the Gene database is still scarce. Curated information can be obtained mainly for mice antisense genes and is explored in several publications (Table S5). On the other hand, the high-throughput sequencing projects in plants generated massive datasets that are yet to be characterized in depth. In particular, the 1132 antisense transcripts predicted in plants (Figure S1a and Figure 4) need to be experimentally investigated.

### 3.2. Sugarcane asRNA

Modern sugarcane varieties have a large size (10 Gb) and complex genome which resulted from a historical process of interspecific crossing between several species [144,145,146]. The chromosomes present 8 to 13 copies each resulting in more than 100 chromosomes (107–114) in total [147]. The auto-allopolyploid genome nature challenges genetics and genomics studies when compared to diploid crops [148]. Despite the recent publications of the genome sequencing of *Saccharum spontaneum* [149] and *Saccharum* spp. hybrids [84,150], much remains to be studied about the genes and their expression regulation. The occurrence of NATs in sugarcane is of special interest as they can potentially be responsible for differences in allele-specific expression.

To date, few articles have devoted attention to antisense transcription in sugarcane. Sugarcane transcriptomes were obtained to study sense and antisense expression in different tissues [33,82,151], in circadian regulation [79], and development [33,43]. Additionally, high-throughput data on sugarcane NATs were investigated under drought conditions [44,80]. Using quantitative PCR, Manimekalai et al. [152] and Narayanan et al. [153] detected an increase in the *Mybas* (Myeloblastosis antisense) gene expression in sugarcane under oxidative stress.

Sugarcane putative NATs were identified for the first time from a SAGE library obtained from sample leaves of the 15-month-old field-grown SP80-3280 cultivar [43]. Antisense transcripts were defined based on their putative annotation and inverse matched to a sense SAS or direct matched to an inverted hit-frame SAS. The functional annotation of this SAGE library showed enrichment for photosynthesis and the carbon accumulation process. However, the biological function of the potential NATs was not verified [43].

According to our search, the first experiment specifically designed to address sugarcane NATs used a customized oligo array, including probes designed to quantify the expression levels of antisense transcripts [44]. Almost 12% of the interrogated genes presented antisense expression and in most cases, the SS/AS pairs presented co-expression [44]. The same oligo array was used to study sugarcane gene expression in several conditions [79,80,81,82,83,84,154,155] but the data on antisense expression was not always addressed. The antisense expression was observed in eight experimental data publications (Table 1), which are briefly presented here.

Ferreira et al. [82] investigated the expression profile of leaves and immature, intermediate, and mature internodes from *Saccharum officinarum*, *S. robustum,* and *S. spontaneum* genotypes and the commercial hybrid RB867515. The authors observed antisense expression in all of the evaluated genotypes. Functional annotation revealed the presence of NATs in several carbohydrate metabolism pathways; however, the expression of NATs was more representative in amino acid metabolism pathways.

Hotta et al. [79] studied the circadian sugarcane transcriptome in leaves of the RB855453 variety grown in a greenhouse. Sense and antisense expression were found to be circadian-regulated in different ways. Antisense transcripts tended to peak at subjective dawn and sense transcripts at the subjective middle of the day. Furthermore, circadian-responsive NATs were functionally classified in photosynthesis, carbohydrate metabolism, amino acid metabolism, and genetic information processing pathways.

Dantas et al. [83] studied the circadian sugarcane transcriptome in leaves and upper, maturing, and mature internodes of field-grown plants of the variety SP80-3280. Although the authors did not discuss antisense transcription in their article, we used their published dataset to investigate SS/AS expression.

Our group has also performed multiple experiments to investigate the transcriptional changes of sugarcane plants under drought conditions. The expression of sense and antisense transcripts was detected in the leaves of the SP90-1638 variety after one, three, and five days of water withholding [44] and in leaves and roots from the variety SP80-3280 after four and six days of water withholding followed by two days of rewatering [80]; both experiments were performed under greenhouse conditions. The transcriptomes of the leaf and upper internodes from three different varieties (RB86-7515, RB92-579, and RB85-5536) were investigated under field conditions without watering [80]. Lembke et al. [44] observed a time course increase in the detection of antisense transcripts in sugarcane samples subject to drought compared to irrigated samples. After one day, the number of antisense probes with significant expression was the same in the non-irrigated and control samples. Three days of treatment resulted in a slight increase in antisense detection in the drought samples. However, in five days, antisense transcription was almost three times higher in drought than in control samples, suggesting an antisense role in the sugarcane response to dehydration. Antisense expression was confirmed by qPCR for the following genes: *fructose-1,6-bisphosphatase I*, *alpha galactosidase 1*, ATAF1 protein, *photosystem II 10kDa polypeptide*, *photosystem I reaction center subunit V*, *magnesium chelatase subunit*, *ribonuclease,* and nucleolar protein Nop56 [44]. Studying co-expression modules in sugarcane under drought conditions, Diniz et al. [80] identified antisense expression in four SAS classified in the photosynthesis co-expression module (M1) and another four SAS allocated to the serine family amino acid metabolic process (M5). These results suggest the importance of NATs in regulating photosynthesis and amino acid metabolism pathways in sugarcane, as presented by Hotta et al. [79] and Ferreira et al. [82].

Cunha et al. [81] investigated the effects of two ethylene-based growth regulators (ethephon and AVG) on the transcriptional profiles of the leaves and upper internodes of sugarcane variety IACSP95-5000 grown under greenhouse conditions. The authors focused on the sense transcripts; however, antisense expression was also detected and the dataset was investigated here.

Finally, the sugarcane transcriptome (SP80-3280 variety) of leaves and immature, intermediate, and mature internodes was studied during plant development in the field [33,84]. The expression profile of NATs was divergent throughout the sugarcane development in the two growing seasons (“one-year” and “one-and-a-half-year sugarcane”), with an emphasis on antisense transcripts related to the phenylpropanoids and Phe, Tyr, and Trp pathways [33].

**Figure 5:**
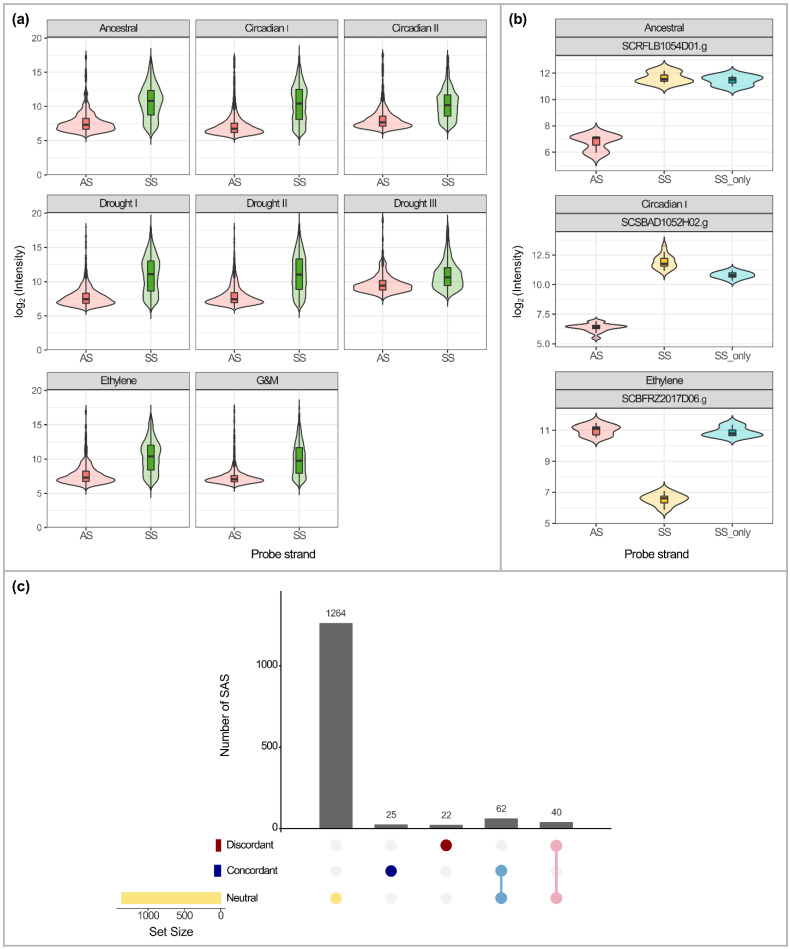
Expression distribution of the sugarcane-assembled sequences (SAS) that presented both sense (SS) and antisense (AS) expression in sugarcane leaves detected in a customized oligo array [44]. The expression of SS/AS pairs was verified in 1413 SAS under different experimental conditions. (**a**) The overall expression of antisense (AS) and sense (SS) transcripts in different experiments: Ancestral, Circadian I, Circadian II, Drought I, Drought II, Drought III, Ethylene, and G and M (Growth and Maturation). (**b**) Examples of three SAS SCRFLB1054D01.g, SCSBAD1052H02.g, and SCBFRZ2017D06.g with concomitant expression of sense (SS) and antisense (AS) transcripts or only sense transcripts (SS_only). SS/AS neutral expression is verified for SCRFLB1054D01.g (Ancestral), whereas concordant and discordant expressions are presented by SCSBAD1052H02.g (Circadian I) and SCBFRZ2017D06.g (Ethylene), respectively. (**c**) Graphic representation of the sugarcane transcript sets, grouped by their SS/AS expression correlation. Yellow: neutral expression correlation. Dark blue: concordant expression. Red: transcripts with a discordant expression pattern. Light blue is the intersection of the sets whose SS/AS expressions are neutral or concordant. Pink: antisense transcript with neutral or discordant expression correlation with its cognate sense. Barplot indicates the total number of SAS in each expression class. Color-filled circles indicate sets of transcripts with expression correlations. Light gray circles: empty classification sets.

For the present review, we took advantage of these multiple experiments performed on the same oligo array platform and investigated the expression level of antisense transcripts. A conservative approach was used to filter the dataset, considering only SAS with significant SS/AS expression in all biological replicates, and a total of 1413 unique SAS was identified. A higher number of SS/AS-expressed pairs was observed in the Circadian I (729) followed by the Growth and Maturation (559) experiments (Table 3). Otherwise, fewer SS/AS pairs were detected in the Drought III (48) and Ancestral (56) experiments (Table 3). We observed that the overall mean expression of sense transcripts was higher than the expression of the antisense transcripts in all experiments (Figure 5a). A similar trend was previously found in sugarcane plants subjected to drought [44].

However, the expression level for each gene can be diverse regarding the presence or absence of the cognate antisense transcript and the experimental conditions. Here, three scenarios were observed in each experiment: (i) the average expression of the sense transcript decreased when the antisense transcript was concomitantly expressed (n = 22); (ii) the average expression of the sense transcript increased when the antisense transcript was expressed (n = 25); and (iii) the average expression of the sense transcript was not significantly altered when the antisense transcript was expressed (n = 1264). The first, second, and third scenarios were, respectively, exemplified in the Ethylene, Circadian I, and Ancestral experiments (Figure 5b). Most SAS with SS/AS expression herein identified presented a neutral expression profile, i.e., sense and antisense expressions were non-correlated. However, concordant (positive correlation) and discordant (negative correlation) expressions were also verified (Table 3).

SS/AS pairs differentially expressed in sugarcane leaves under drought exhibited a predominance of positive correlations, although some negatively-correlated or without-correlation pairs were detected [44]. Similar results were found in *Arabidopsis*, whose antisense mostly showed concordant expression to the cognate genes; however, some had a neutral or discordant expression [21].

The requirements for statistical analysis may have influenced the proportion of genes with neutral expression herein identified, because some genes with a putative significant correlation (positive or negative) could not be analyzed. In situations where all biological samples presented SS and AS expression, we did not have the average value of only the SS expression for comparison. Likewise, when just one sample showed simultaneous SS/AS or only SS expression, the mean expression values could not be calculated and the analysis disregarded the cognate SAS.

A biological explanation for the high number of SAS with uncorrelated SS/AS expression was proposed by Hotta [79]. The authors used Spearman’s rank correlation coefficient to investigate the expression patterns of circadian-rhythmic SS/AS pairs and detected a bimodal distribution. These results suggested two regulatory mechanisms, one that affects both SS and AS expression and another in which AS expression is independent of its cognate SS [79].

Considering the SAS with a positive SS/AS correlation, only 8% (2/25) showed the same expression profile in the different experiments. SCCCAM2003F01.g showed concordant expression in Circadian I and II, whereas SCJFRZ2013F03.g had a positive SS/AS correlation in Circadian I and Drought I. The remaining SAS with a concordant expression were detected in a unique experiment (Table S8). Similarly, only 18% (4/22) of discordant SS/AS pairs were observed in more than one experiment. SCRFHR1006H10.g had the most frequent discordant expression, identified in the Circadian I, Circadian II, and Ethylene experiments. Discordant expression was detected twice for SAS SCSBAD1087B08.g, SCJLRT1006H10.g, and SCCBSD2038H10.g (Table S8).

Comparing the concordant/discordant ratio in the same experiment, we can highlight Drought II, which presented ten times more concordant SS/AS pairs. In contrast, in the G and M experiment, the discordant pairs were nine times more frequent (Table 3).

When all experimental data were investigated, it was observed that the same sense–antisense pairs may present concordant, discordant, or neutral expression, depending on the experiment (Figure 5c; Table S8). This could reflect the different mechanisms of antisense function and sense transcription regulation.

Another question regarding antisense transcription in polyploid genomes is if the transcription occurs in all homo(eo)logs or if the expression regulation among homo(eo)logs is different. The sugarcane ORFeome sequencing and the genome assembly to a copy-resolved gene space revealed that few of the homo(eo)logs were transcribed in the antisense orientation [84,151]. Differences in the regulatory sequences among the homo(eo)log promoter regions (upstream to transcription starting site—TSS) may explain the differences in antisense transcription regulation [84]. In *Brassica napus* and *Gossypium barbadense*, two polyploid species, the expression of lncRNA, including the long noncoding natural antisense transcript (lncNAT), was different among the two homoeologous subgenomes [156,157]. Additionally, the detection of antisense sequences with high expression levels seemed to be strongly influenced by the sequencing methodology. Shen et al. [157] found lncNAT more expressed than their cognate mRNA when transcription levels were analyzed in *Brassica napus* by rRNA-depleted RNA seq, but the inverse expression pattern was observed using poly(A) RNAseq.

The results discussed here reveal that the study of NATs is promising in gene expression investigation in sugarcane. However, more conclusive data on SS/AS expression patterns depends on experiments specifically designed for this purpose.

## 4. Conclusions

Regulatory RNAs, such as NATs, represent much of the complexity of gene expression and are widely distributed in eukaryotic genomes including plants. NATs can modulate gene expression, acting on epigenetic, pre-, or post-transcriptional regulation in response to biotic, abiotic, and developmental stimuli. Studies on natural antisense in plants are still scarce and more focused, especially on cis-NAT and model plants. On the other hand, artificial antisense in Genetic Engineering, widely used in gene silencing, has been less addressed in publications in the last five years. Several transcriptomic approaches have been used to study asRNA in plants; however, different techniques can present different results of the antisense expression. It is essential to consider the type and cellular localization of the target asRNA before choosing the identification methodology. Transcriptional data from sugarcane show the occurrence of NAT in different growth conditions, varieties, tissues, and treatments. Sugarcane NATs with concordant, discordant, or neutral expression with sense cognate genes were identified. It is suggested that the antisense transcript plays a role in regulating homo(eo)logs’ differential expression. The efforts to study NATs in plants should cover the identification of known and new NATs, their mechanisms of action, and possible functional roles, shedding light on and providing insights into the knowledge of model plants to be tested on non-model plants. This is especially noted in the case of cultivated plants with polyploid genomes. The differences in the expression of antisense homo(eo)logs already observed in polyploid genomes add an extra layer to the expression regulation complexity.

## Figures and Tables

**Table 1:** Public oligo array datasets using a customized array for quantifying gene expression in both sense and antisense orientations in sugarcane.

GEO Series	Experiment ID	Experiment Description	Number of Hybridizations	Reference
	Ancestral	Sugarcane ancestral genotypes: *Saccharum officinarum*, *S. spontaneum,* and *S. robustum*	36	Ferreira et al. [82]
GSE42725	Circadian I	Circadian rhythms of sense and antisense transcriptions	22	Hotta et al. [79]
GSE129543	Circadian II	Vast differences in organ-specific rhythms of transcription in field-grown sugarcane	84	Dantas et al. [83]
GSE33574	Drought I	Sugarcane expression data from stress time series	6	Lembke et al. [44]
GSE125070	Drought II	Sugarcane drought experiment under field conditions	12	Diniz et al. [80]
GSE125069	Drought III	SP80-3280 drought and rewatering	12	Diniz et al. [80]
GPL22278	Ethylene	Ethephon- and AVG-induced transcriptional changes	48	Cunha et al. [81]
GSE124990	G and M ^1^	SP80-3280 growth and maturation under field conditions	30	Souza et al. [84]; Wijma et al. [33]

^1^ G and M: Growth and Maturation.

**Table 2:** List of the antisense sequences of plants and their cognate sense genes, available at Gene database (NCBI) on 18 February 2022. All these antisense genes are associated with citations on PubMed.

Antisense-Related Gene				Cognate Sense Gene				
Gene ID	Gene Symbol	Gene Type	Citations ^1^	Gene ID	Gene Symbol	Gene Type	Citations ^1^	Species
5008140	*AT4G13505*	ncRNA	1	826983	*AMT1;1*	protein-coding	18	*Arabidopsis* *thaliana*
5008149	*AT4G20362*	ncRNA	1	827784	*RABE1b*	protein-coding	30	*Arabidopsis* *thaliana*
6241025	*AT1G69572*	ncRNA	1	843293	*AT1G69570*	protein-coding	7	*Arabidopsis* *thaliana*
28720403	*AT5G01675*	ncRNA	3	830878	*FLC*	protein-coding	142	*Arabidopsis* *thaliana*
109115966	*lncRNA16397*	ncRNA	1	101250592	*GRX22*	protein-coding	2	*Solanum* *lycopersicum*
109461486	*LOC109461486*	ncRNA	1	100284505	*LOC100284505*	protein-coding	4	*Zea* *mays*
5008210	*MIR398b*	ncRNA	7	831306	*AT5G14550*	protein-coding	3	*Arabidopsis* *thaliana*
28721157	*MIR398c*	miscRNA	4	831308	*NRT2.7*	protein-coding	5	*Arabidopsis* *thaliana*
104795691	*MIR398*	ncRNA	3	103856086	*LOC103856086*	protein-coding	0	*Brassica* *rapa*

^1^ Number of citations in PubMed until 18 February 2022.

**Table 3:** Total of sense/antisense (SS/AS) pairs and the expression pattern detected in each investigated experiment.

SS/AS Expression	Ancestral	Circadian I	Circadian II	Drought I	Drought II	Drought III	Ethylene	G and M ^1^
Concordant	0	41	21	3	21	1	4	1
Discordant	1	37	12	5	2	0	9	9
Neutral	55	651	225	295	186	47	136	549
Total	56	729	258	303	209	48	149	559

^1^ G and M: Growth and Maturation.

## Data Availability

Not applicable.

## Supplementary Materials

The following supporting information can be downloaded at: https://www.mdpi.com/article/10.3390/ijms231911603/s1.
