# Supplementary material for: Antisense Transcription in Plants: A Systematic Review and an Update on cis-NATs of Sugarcane
Source: Int J Mol Sci. 2022 Oct 1;23(19):11603. doi: 10.3390/ijms231911603 (PMC9569758; doi:10.3390/ijms231911603)
Supplement: Supplementary file 1 [file ijms-23-11603-s001.zip › Figure S1.pdf]

International Journal of
Molecular Sciences

MDPI

Review

Antisense Transcription in Plants: A Systematic Review and an Update on cis-NATs of Sugarcane

Luciane Santini[1], Leonardo Yoshida[1], Kaique Dias de Oliveira[1], Carolina Gimiliani Lembke[1], Augusto Lima Diniz[1], Geraldo Cesar Cantelli[1], Milton Yutaka Nishiyama-Junior[2], Glaucia Mendes Souza[1],*

1 Departamento de Bioquímica, Instituto de Química, Universidade de São Paulo, São Paulo, 05508-900, Brazil
2 Laboratório de Toxinologia Aplicada, Instituto Butantan, São Paulo, 05503-900, Brazil
* Correspondence: glmsouza@iq.usp.br

Supplementary Figure S1

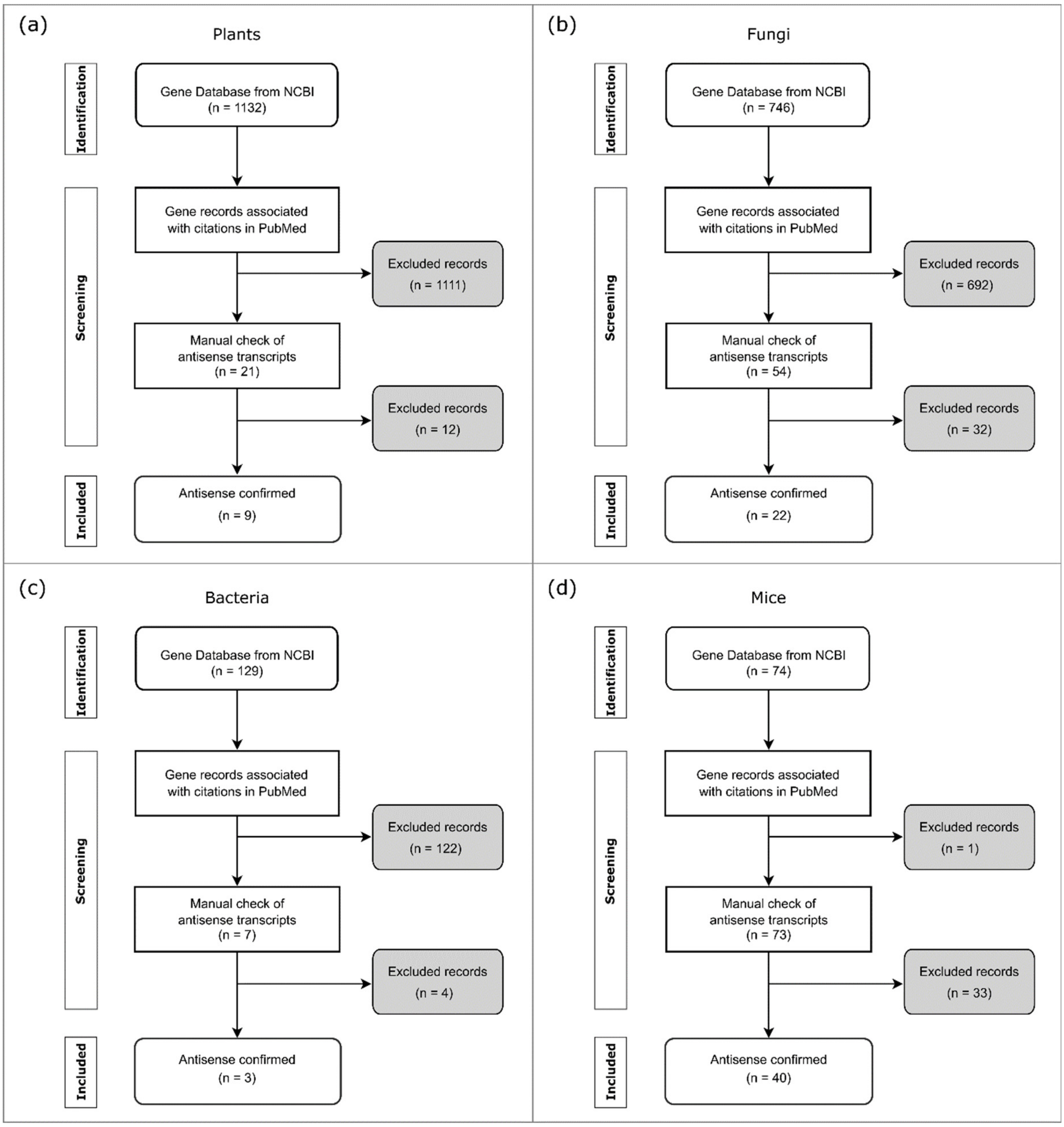

Figure S1. Workflow and results of the systematic search for gene sequences related to antisense expression in plants (a), fungi (b), bacteria (c), and mice (d). White: screening process; gray: excluded records. The search was conducted on the "Gene" database from NCBI (https://www.ncbi.nlm.nih.gov/) on 18 February, 2022.
